# Fire (plus) flood (equals) beach: coastal response to an exceptional river sediment discharge event

**DOI:** 10.1038/s41598-022-07209-0

**Published:** 2022-03-09

**Authors:** Jonathan A. Warrick, Kilian Vos, Amy E. East, Sean Vitousek

**Affiliations:** 1 U.S. Geological Survey, Santa Cruz, CA USA; 2 Water Research Laboratory, School of Civil and Environmental Engineering, University of New South Wales, Sydney, Australia; 3 University of Illinois at Chicago, Chicago, IL USA

**Keywords:** Geomorphology, Environmental health, Natural hazards

## Abstract

Wildfire and post-fire rainfall have resounding effects on hillslope processes and sediment yields of mountainous landscapes. Yet, it remains unclear how fire–flood sequences influence downstream coastal littoral systems. It is timely to examine terrestrial–coastal connections because climate change is increasing the frequency, size, and intensity of wildfires, altering precipitation rates, and accelerating sea-level rise; and these factors can be understood as contrasting accretionary and erosive agents for coastal systems. Here we provide new satellite-derived shoreline measurements of Big Sur, California and show how river sediment discharge significantly influenced shoreline positions during the past several decades. A 2016 wildfire followed by record precipitation increased sediment discharge in the Big Sur River and resulted in almost half of the total river sediment load of the past 50 years (~ 2.2 of ~ 4.8 Mt). Roughly 30% of this river sediment was inferred to be littoral-grade sand and was incorporated into the littoral cell, causing the widest beaches in the 37-year satellite record and spreading downcoast over timescales of years. Hence, the impact of fire–flood events on coastal sediment budgets may be substantial, and these impacts may increase with time considering projected intensification of wildfires and extreme rain events under global warming.

## Introduction

Wildfire is an important factor in hillslope erosion and sediment yields of mountainous and forested rivers worldwide^1,2^. Fires liberate soil materials from hillslopes through dry ravel, inhibit rainwater infiltration of soils, and increase the likelihood of soil erosion during subsequent rainfall, all of which increase sediment transport to and through rivers. Yet not all wildfires induce similar geomorphic responses. Factors that control post-fire responses include soil burn severity, basin slope, post-fire rainfall intensities and timing, and downslope floodplain morphology^1–4^. Climate change is increasing the size, intensity, and frequency of wildfires throughout the world^5,6^, while concurrently increasing the likelihood for extreme rainfall such that burned areas are at greater risk of experiencing rain intensities exceeding thresholds for debris flow and other sediment mobilization processes^7–9^. These evolving conditions are expected to increase fire-related sediment yields and alter downstream riparian habitats in the future^10–12^.

Although there is increased understanding of the role of wildfire in terrestrial and fluvial systems, little is known about wildfire’s role on downstream coastal landforms. Several challenges exist in linking upslope processes to coastal morphodynamics, including the infrequent nature of wildfire (recurrences are historically 10–100 years, although frequency is expected to increase in warmer climates^1,5,12^), the complexities and variability of sediment transport through wildfire-affected landscapes^1,3,13–15^, the inherent variability of beach widths due to oceanic processes^16^, and the general scarcity of shoreline position and beach topographic monitoring to determine coastal effects^17,18^. Although both geologic records and the coastal waters, sediments, and ecosystems near burned watersheds provide evidence that wildfires influence sediment delivery to the coast^19–22^, there is no general geomorphic understanding about the role of periodic sediment production from wildfire on coastal landforms.

Coastal landforms throughout the world are encountering multiple stressors such as accelerating sea-level rise and modified sediment budgets^23–26^. Thus, a better understanding of fluvial sediment inputs to beaches, marshes, deltas, and other coastal landforms is needed^27–30^. Wildfire is not the only factor influencing fluvial sediment discharge to the coast. Other human impacts such as agricultural development and dam construction have modified rates and characteristics of fluvial sediment discharge for decades to millennia, and coastal impacts from these changes are especially evident in the world’s larger deltas^24,27,31–35^. However, as wildfire can dictate watershed sediment discharge, particularly for small, steep coastal watersheds^1,4,12^, it deserves more attention with respect to coastal sediment budgets and the future vulnerability of coastal landforms. The development of new tools to map shorelines from satellites over intervals of decades^18,36^ provides new opportunities to evaluate time-dependent coastal impacts of events such as wildfires.

**Figure 1:**
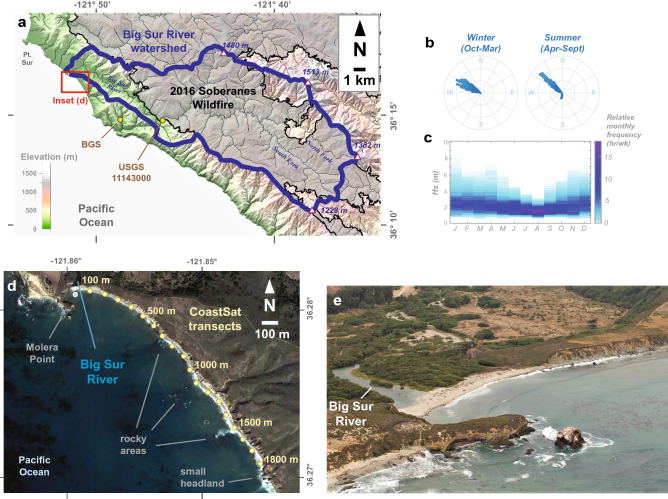
Location and background information of the Big Sur, California study area. (**a**) Watershed map of the Big Sur River watershed, including the boundaries of the 2016 Soberanes wildfire and sampling stations. Map was created in Global Mapper version 21.1.0 (https://www.bluemarblegeo.com/) with data from the USGS 3D Elevation Program, USGS National Watershed Boundary Dataset, USGS National Hydrography Dataset, and the California Department of Forestry and Fire Protection's Fire and Resource Assessment Program (FRAP). (**b**,**c**) Offshore wave climatology from the offshore Cape San Martin buoy (Station 46028; 35.770° N 121.903° W). (**d**) Inset map of the Big Sur River mouth and littoral cell study area, including the eighteen CoastSat transects monitored for this study. Map was created from Google Earth Pro version 7.3.4.8248 imagery and data (https://www.google.com/earth/versions/) and assembled in Abobe Illustrator version 26.0.2. (**e**) An oblique aerial photo of the Big Sur River mouth and approximately 500 m of the study area beach from Sept. 11th, 2015, when the study area beach was relatively narrow compared to historical conditions (photo credit: Kenneth and Gabrielle Adelman, California Coastal Records Project, www.Californiacoastline.org).

Here we address several questions about wildfire and coastal littoral systems: what is the role of wildfire in a coastal sediment budget? How does a littoral cell respond to large inputs of sediment related to wildfire? Do all wildfires influence the coast in similar ways? Can new remote-sensing techniques be used to fill critical coastal data gaps, especially for infrequent sediment input events? We address these questions by applying new satellite remote sensing techniques^18,36^ to the steep and fire–prone region of Big Sur, California (Fig. 1), an area with important ecological and recreational value but negligible in situ coastal monitoring.

### Sources of sediment

The Big Sur River drains a steep watershed incorporating 151 km^2^ of the Santa Lucia Range of California and is a primary source of coastal sediment in the region^37^ (Fig. 1). The watershed boundary lies along ridges that are generally 1200 m to over 1500 m in elevation, and the river discharges into the Pacific Ocean roughly 5 km downcoast (south) of Point Sur (Fig. 1a). Only 2% of the watershed area is classified as developed, which includes privately owned campgrounds and a highway transportation corridor^38^. Land cover is dominated by forests with redwood or oak overstory, chaparral, and grasslands, all of which are fire prone during the dry California summers^38^.

**Figure 2:**
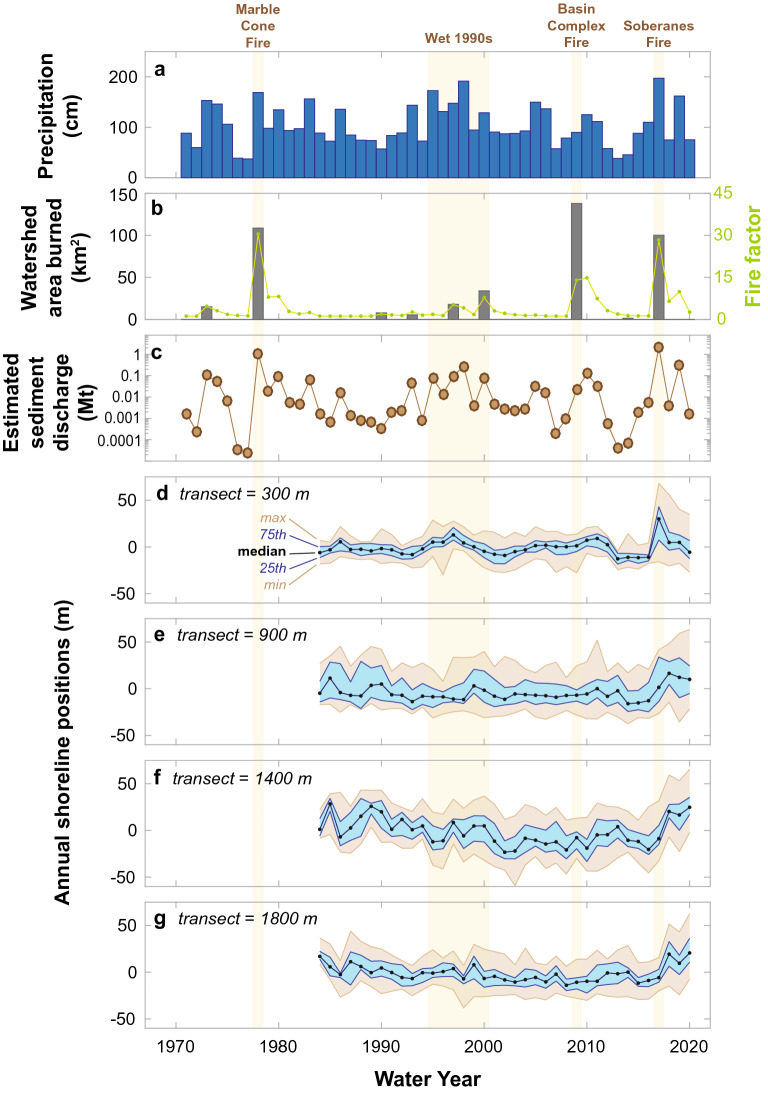
Annual observations of the Big Sur River mouth study area summarized over water year increments of time (Oct 1–Sept 30). (**a**) Annual precipitation at the Big Sur State Park (BGS) sensor. (**b**) Annual area of the Big Sur River watershed burned by wildfire (grey bars) and the resulting Fire Factor that defines the magnitude of sediment discharge increase from the combined effects of fire and precipitation (green symbols). (**c**) Estimated annual suspended–sediment discharge from the Big Sur River, symbol size is proportional to the 1-σ lognormal variance of each estimate. (**d**–**g**) Shoreline positions for 4 of the 18 CoastSat transects summarized by annual statistics, including the median, 25th and 75th percentiles, and the minimum and maximum. Shading highlights the interquartile range (blue) and the total range (brown) for each water year. A complete set of annual plots for all transects is provided in the Supplemental Materials.

Wildfire is common in the Big Sur River watershed and the surrounding mountains owing to steep landscapes, dry summer climate, and mixed vegetation ground cover^4,38^. For example, the 2016 Soberanes wildfire burned 537 km^2^ of the Santa Lucia Range, approximately 100 km^2^ of which were within Big Sur River watershed (Fig. 1a)^39^. This wildfire burned the headlands of the watershed at moderate to high soil burn severity, resulting in near complete incineration of organic materials and the development of water-repellent layers in soils^39^. Two other large historical wildfires occurred in the Big Sur River watershed: the 1977 Marble Cone and 2008 Basin Complex fires, which burned 109 and 138 km^2^, respectively, of the Big Sur River watershed (Fig. 2b; see “Methods”).

Estimates of sediment discharge from the Big Sur River, which has received negligible sediment monitoring, were generated by transferring detailed pre- and post-wildfire records from the adjacent and similar Arroyo Seco watershed^4^ (see “Methods”). These techniques used a simple precipitation-regulated fire response function, the non-dimensional Fire Factor (*F*_*f*_), which was derived empirically and represents the scale of fire-related effects on sediment yields. Values of *F*_*f*_ exceeded 30 following both the 1977 Marble Cone and 2016 Soberanes wildfires (Fig. 2b), and the estimated 1.1 and 2.2 Mt (10^6^ tonnes) of sediment discharge, respectively, during these years combine to roughly 70% of the 50-year sediment discharge (4.8 Mt; 1971–2020; Fig. 2c). These two wildfire-affected winters (1977–1978 and 2016–2017) had greater sediment discharge than following the 2008 Basin Complex wildfire (0.18 Mt over three years) or the 1995–2000 wet interval (0.54 Mt over 6 years; Fig. 2a–c), which was similarly noted in regional studies of these events^4,40^. The increased sediment discharge in 2016–2017 was due in part to record-setting rainfall along coastal California in early 2017, when atmospheric vapor flux to the coast was three standard deviations above normal due to unusual atmospheric-river activity^41^, causing repeated flooding and large increases in regional sediment flux to the coast^42^. During 47 days of January–February 2017 the Big Sur River experienced six peak flows that exceeded the 2-year flood magnitude, including two near the 10-year flood levels: January 10th (144 m^3^/s) and February 20th (140 m^3^/s; see “Methods”). For comparison, the wet season immediately following the 1977 Marble Cone wildfire included the largest recorded flood of the 50-year record (303 m^3^/s), and the winter after the 2008 Basin Complex wildfire had no flows above the 2-year flood magnitude.

Another sediment source to the study area beach is erosion of the sea cliffs south of the river mouth (Fig. 1e). Cliff erosion is approximately 0.3 m/year along the study area^43^, which suggests that approximately 12,500 m^3^/year (~ 25,000 t/year) of cliff erosion occurs from the 1700 m long and ~ 25 m high mixed bedrock and unconsolidated sediment sea cliffs (see “Methods”). Thus, cliff erosion is approximately 4-times less than the average river sediment input, although it may be a primary sediment source during multi-year droughts, and it is the source of several boulders along the coast (Fig. 1d,e).

### Shoreline responses following the 2016 wildfire

The Big Sur River mouth study area is immediately downcoast (south) of Molera Point headland, and it includes a narrow sandy beach, several areas with rocky nearshore morphology (~ 700, 1000, and 1500 m from the river mouth), and a small headland ~ 1900 m from the river mouth (Fig. 1d,e). The shoreline shape is influenced by these geomorphic features, including shoreline curvature for ~ 500 m downcoast of Molera Point and ~ 10–30 m seaward extension of the shoreline at the rocky areas (Fig. 1d).

Time series of shoreline change between 1984 and 2020 were obtained from the CoastSat toolbox^36^, which maps shoreline positions from publicly available Landsat and Sentinel-2 imagery. Mapped shorelines of the study area were transferred to eighteen 100-m spaced cross-shore transects and corrected for tidal stage during each image (Fig. 1d; see “Methods”). We have also generated similar transect data for adjacent coastal areas for comparative purposes, as discussed below and presented fully in the Supplemental Materials.

Each transect has over 1000 shoreline measurements during the 37-year satellite record, and these observations were summarized into annual shoreline metrics for Fig. 2d–g (all transects plotted in Supplemental Materials). Overall, transects near the northern and southern ends of the study area have lower annual variability as shown by the interquartile range (blue shading; Fig. 2d–g). This is related to wave-driven seasonality in the shoreline, which is strongest in the central portion of the study area. Perhaps the most distinct patterns in the shoreline data, however, are the accretion patterns that occurred during and after 2017 (Fig. 2d–g). During the 2017–2020 interval, all transects recorded either the most seaward median and quartile shoreline positions of the entire record or the most seaward positions since 1984. The timing and extent of beach widening differed across the study area, however, as the transects adjacent to the river mouth narrowed significantly after 2017, whereas the downcoast transects continued to widen through 2020 (Fig. 2d–g).

**Figure 3:**
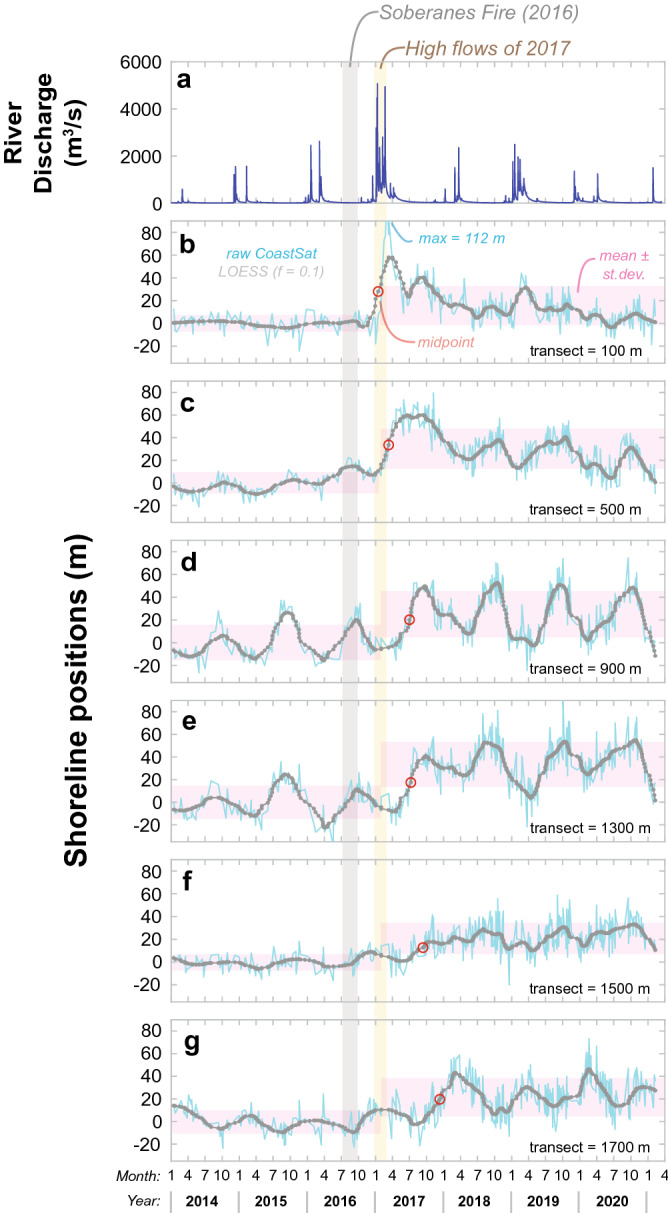
Detailed comparisons of Big Sur River discharge and CoastSat shoreline positions for six transects in the study area highlighting the years immediately before and after the 2016–2017 fire–flood event. The timing of the Soberanes fire and the highest river flow rates are highlighted with vertical bars. (**a**) Instantaneous river discharge from 15-min observations at the Big Sur River at Big Sur gauge (USGS streamgage 11143000). (**b**–**g**) Shoreline positions for six transects, including the raw CoastSat observations (blue lines), a LOESS-smoothed function through these observations (grey lines), and the mean ± st. dev. of the raw shoreline positions during the intervals before and after the peak in water discharge (pink boxes; see “Methods”). Also shown are the timing of the midpoint of the initiation of accretion (red symbols; see “Methods”). Note that the widest observation for the 100 m transect was off the figure scale. A complete set of plots for all transects is provided in the Supplemental Materials.

Raw shoreline measurements provide additional details about the timing and extent of the 2017–2020 beach widening (Fig. 3). Accretion near the river mouth did not occur until after the high river discharge events of 2017, several months after the 2016 Soberanes wildfire (Fig. 3a,b). By March 2017 the shoreline adjacent to the river mouth had accreted over 100 m, but this accretion was ephemeral as the shoreline retreated soon afterward due to longshore spreading of sediment (Fig. 3b). The downcoast shoreline exhibited several different patterns following the 2016–2017 fire–flood event including: (1) lower magnitude and delayed accretion in the shoreline immediately downcoast of the river mouth (e.g., Fig. 3c) and (2) general widening of the beach with an increase variability—including seasonality—at the middle to southern transects (Fig. 3d–g; all transects plotted in Supplemental Materials). The average accretion in the shorelines over the 2017–2020 interval ranged between 15.2 and 33.3 m (mean = 23.2 m) and variance in the shoreline also increased after the fire-flood event as shown by red boxes in Fig. 3. The increase in mean beach width for all 18 transects were found to be highly statistically significant (*p* < 0.0001) using Welch’s *t*-tests (see “Methods” for details).

**Figure 4:**
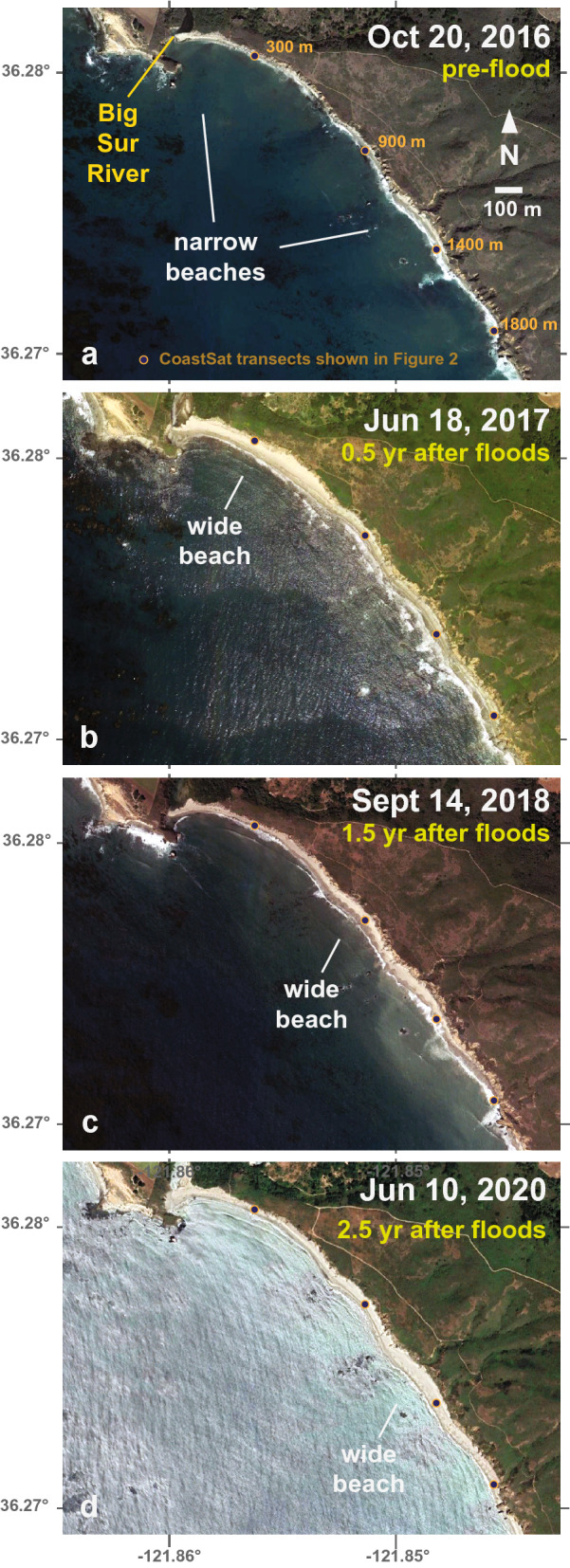
Imagery of the Big Sur River mouth and adjacent beaches (**a**) before and (**b**–**d**) after the 2016–2017 fire–flood events. The approximate location of the widest beach is highlighted by text in each image, and the location of transects presented in Fig. 2 are shown with symbols and labeled in (**a**). Maps were created from Google Earth Pro version 7.3.4.8248 imagery and data (https://www.google.com/earth/versions/) and assembled in Abobe Illustrator version 26.0.2.

Higher spatial resolution satellite imagery provides additional information about shoreline patterns after the 2016–2017 fire–flood event. Immediately after the 2016 Soberanes wildfire, but before the high river flows of early 2017, the beaches of the study area were relatively narrow (Fig. 4a). These conditions coincided with one of the strongest multi-year droughts in California’s precipitation records (2012–2015) and some of the narrowest beach conditions of the 1984–2020 satellite-derived shoreline records (Fig. 2). However, after the high river flows of early 2017, a wider sandy beach was observed in the upcoast (northern) portion of the study area (Fig. 4b), which extended south with time (Fig. 4c,d). This imagery was obtained during summer to early fall seasons when beach widths were generally greatest (cf. Fig. 3). Thus, there is evidence from the transect data and higher-resolution imagery that beach progradation started near the river mouth and spread downcoast (south) over time (Figs. 3, 4).

### River sediment supply hypothesis

To examine the hypothesis that the sediment comprising the new beach widening in 2017 was derived from the Big Sur River, we evaluated three elements: a simple sediment budget, patterns in remote sensing imagery, and CoastSat data from other regional beaches without river sediment inputs. First, a sediment budget was derived from estimates of river discharge and beach sedimentation. As noted above, the 2016–2017 suspended-sediment discharge was estimated to be 2.2 Mt. Only a portion of this sediment was coarse enough to be compatible with the littoral cell, as finer-grained sediment will be transported offshore^44^. The sand content (> 63 µm) of the post-fire river discharge was estimated to be 67.5% from the two highest discharge rates sampled by the USGS for suspended-sediment in the Arroyo Seco following the Marble Cone wildfire. Limber et al.^44^ suggested only approximately 45% of this total sand will be coarser than the typical littoral cutoff diameter of 120-µm for California rivers and beach settings. Thus, only ~ 30% of the total sediment discharge, or approximately 0.66 Mt, was estimated to have compatible grain sizes with the littoral cell. Assuming a bulk density of 1500 kg/m^3^, an estimated 440,000 m^3^ of beach-compatible sand was discharged by the Big Sur River during the 2016–2017 winter.

The total sedimentation within the littoral cell was estimated by assuming that the new sand uniformly covered the littoral profile from the upper beach (~ 2 m elevation) to the estimated depth of closure (~ 13 m water depth from standard formulations^45,46^). By May 2017, there was ~ 50 m of progradation along 600 m of the beach and using the full-profile assumption across the 15 m of the active littoral height, the total volume of new beach sand was estimated to be 450,000 m^3^. Although the two elements of the sediment budget differ by only ~ 2%, there is likely up to 50% uncertainty in each value. Regardless, the simple sediment budget suggests that the volume of sand discharged by the river and the volume of new beach sand are of the same magnitude, thereby allowing for the possibility of river sediment causing the coast changes.

**Figure 5:**
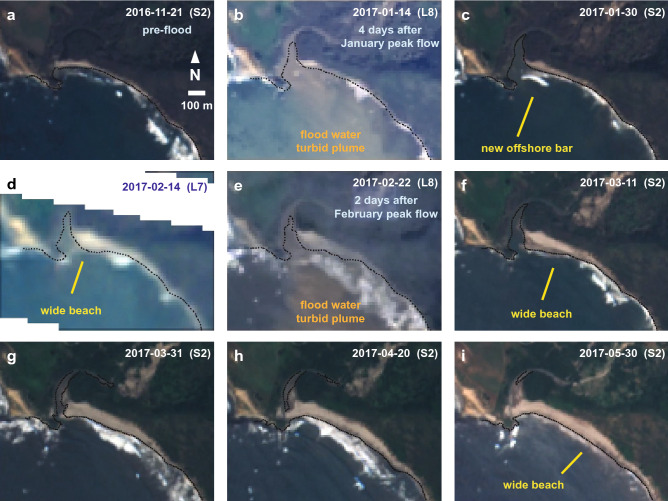
Sentinel-2 (S2) and Landsat (L7 and L8) imagery of the Big Sur River mouth highlighting coastal changes from river sediment contributions during the winter and spring of 2016–2017. Images are top-of-atmosphere true color (RGB) products and include the mapped shoreline position from the CoastSat algorithms (black lines). Imagery was extracted for areas bounding 36.2762° N to 36.2845° N latitude and − 121.8497° to − 121.8609° longitude. The image from Landsat-7 shown in (**d**) include scanline errors that are shown as no data (white areas). Imagery were obtained through Google Earth Engine (https://earthengine.google.com/) using the CoastSat tool box version 1.1.2 (https://github.com/kvos/CoastSat) and assembled in Abobe Illustrator version 26.0.2.

Observations of imagery from the Sentinel-2 and Landsat sensors provide additional support for a river source of sediment (Fig. 5). This imagery clearly shows negligible shoreline change after the largest and most sustained high flows in the river during January 2017 (Fig. 5a–c). However, several weeks after the high river discharge, a new bar was observed immediately offshore of the river mouth as seen from wave breaking (Fig. 5c), and several weeks later the beach showed accretion near the location of the former bar (Fig. 5d). The final pulse of flooding in February 2017 did not coincide with additional beach accretion (Fig. 5e), although accretion was measured during the weeks after this event (Fig. 5f,g). These observations are consistent with observations and theory of how river sand is integrated into littoral cells: sand deposition initially occurs in the submarine region offshore of the river mouth and is reworked by waves toward land to form a subaerial beach as the beach profile is reestablished^47,48^.

Lastly, it is valuable to examine other beaches in the region to see if they responded similarly to the Big Sur River mouth. Two beaches immediately north of the Big Sur River have no direct river sediment inputs and were chosen for this task. A complete presentation of this comparison is provided in the Supplemental Materials. We found that unlike the Big Sur River study area, the two beaches did not show widening during the fire–flood interval of 2017–2020. Those beaches also had correlation patterns with sediment inputs that were inverse to those by the Big Sur River mouth. In total, these factors suggest that the Big Sur River mouth shoreline exhibited different temporal patterns to regional beaches. Combined these results support a conclusion that fluvial sediment inputs were responsible for beach widening near the Big Sur River mouth.

### Alongshore sediment spreading

**Figure 6:**
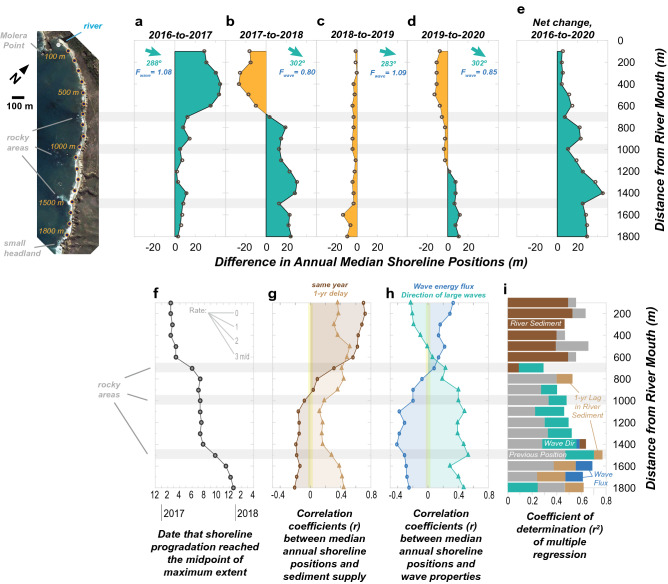
Summary measurements of the shoreline response to the 2016–2017 fire–flood event and the shoreline patterns and regression results from the Big Sur River study site. A rotated map of the study area is shown in the upper left for reference. (**a**–**d**) Year-to-year differences in the annual median CoastSat shoreline positions showing areas of accretion (green) and erosion (orange). Also shown are the annual wave parameters (*F*_*wave*_ and *Dir*; see “Methods”) for the fall-to-winter storm seasons between the two years. (**e**) The net change during 2016–2020 from differences in annual median shoreline positions. (**f**) Timing of the initiation of shoreline accretion as shown by the date for which the shoreline position reached the midpoint of the maximum accretion (see Fig. 3 and “Methods”). (**g**–**h**) Correlation coefficients (*r*) for single variable regressions between annual median shoreline positions and the log_10_-transformed river sediment discharge values (both the same year and 1-year lagged) and the storm-season wave parameters. (**i**) Stepwise multiple variable regression results showing the total *r*^2^ for the net result (total bar length), the variables contributing significantly to this regression with *p* < 0.05 (bar colors), and the stepwise total *r*^2^ for each added variable (bar increment lengths).

Once in the littoral cell, sediment spread from the river mouth toward the south. To examine this spreading of sediment, changes in the 2016-to-2020 annual median shoreline positions were compared for each transect (Fig. 6a–e). These data reveal that the largest shoreline responses during 2016–2017 were within 600 m of the river mouth, where median accretion was 29–45 m (Fig. 6a). However, this same area exhibited 10–27 m of erosion in the subsequent year, while the remaining transects south of 600 m experienced up to 31 m of accretion (Fig. 6b). Thus, the annual shoreline position data provide evidence that accretion started near the river mouth and spread downcoast over the following year. After tens of meters of shoreline change during the first two years, annual shoreline change was less during the subsequent years (Fig. 6c,d). These changes included 2018-to-2019 erosion and 2019-to-2020 rotation, each of which occurred under different storm-season (Oct.–Mar.) wave conditions. Overall, the net shoreline changes between 2016 and 2020 revealed that accretion was greatest toward the southern portion of the study area, where up to 45 m of accretion occurred by 2020, not the river mouth (Fig. 6e).

Downcoast sediment spreading was also evaluated by arrival dates of measurable accretion at each transect. Because accretion occurred over intervals of weeks to years, we took an approach of identifying the initial dates of specific proportions of the maximum progradation (see “Methods”). This technique revealed that the initiation of accretion started next to the river mouth and proceeded systematically downcoast (red circles; Fig. 3b–g). Accretion initiation was clearly related to seasonal shoreline cycles, especially for transects in the middle of the study area. A compilation of these data for all transects reveals three distinct accretion intervals: (1) an initial interval during early 2017 near the river mouth (100–600 m), (2) a relatively uniform interval in summer 2017 for the middle of the study area (700–1400 m), and (3) the Sep.–Dec. 2017 accretion along the most downcoast region (1500–1800 m; Fig. 6f). These measurements suggest an arrival time of ~ 310 days between the river mouth and the 1800-m transect (equivalent to an average speed of 5.8 m/day). We hypothesize that the near uniform initial arrival of sediment in the central area may be related to sediment spreading to this region within the shallow subtidal regions and onshore transport during the seasonal widening of the beach.

Thus, shoreline positions provide evidence that sediment was introduced at the river mouth in early 2017 and spread downcoast in a manner that was influenced by the morphology of the coast, the wave conditions, and the seasonality of shorelines. Thus, large river sediment inputs can influence local and downcoast shoreline widths for many years as dictated by the littoral transport. In the next section we use records of sediment discharge and wave conditions to evaluate how these factors are related to the region’s shoreline positions over decadal scales.

### Evaluation of factors influencing shoreline positions

To examine factors influencing shoreline positions near the Big Sur River, correlation analyses were made with annual measurements of sediment discharge and wave conditions for the coincidental 1984–2020 records (see “Methods”). Correlations between these variables and shoreline positions were generally weak, however, suggesting that no one external variable explained the majority of variance in the shoreline positions (Fig. 6g,h). An exception was found for transects near the river mouth (100–600 m), which had positive correlations (r = 0.56–0.72) with river sediment discharge (Fig. 6g; see Supplemental Materials for additional correlation information). If a delayed response to sediment discharge is considered, for example by including a 1-year lag for the shoreline response, we found weak, positively correlated relationships (r = 0.12–0.52) across the entire study area (Fig. 6g).

Wave effects were examined by considering the wave energy flux during the Oct.-Mar. storm season and the average direction of the most powerful wave conditions of each year, factors that drive shoreline change within the broader region^17^ (see “Methods”). However, these correlations were also weak (r = − 0.41 to 0.33 and − 0.23 to 0.53, respectively), although they did reveal transitions in the sign of correlation at 500–700 m from the river mouth (Fig. 6h). These results suggest that steeper (more northerly) winter wave angles resulted in somewhat narrower beaches in the north and wider beaches in the south. Additionally, the results suggest that higher wave-energy fluxes were generally related to wider beaches in the north and narrower beaches in the south (Fig. 6h).

Although the single-variable correlation results were consistently weak across the study area, they provided evidence that multiple factors contributed to influence shoreline positions. To test this hypothesis, we conducted a stepwise multiple regression for each transect with the variables described above and one additional variable, the preceding year’s shoreline position (see “Methods”). Results suggest that the combination of factors explained an average of 57% (and up to 78%) of the shoreline position variance, which far exceeded the single-variable correlations (Fig. 6i). Sediment discharge was the dominant variable of the northern portion of the study area, whereas the previous shoreline position and wave direction were generally important variables in the middle and southern portion of the study area (Fig. 6i). On the southern end (1500–1800 m), wave energy flux and the 1-year lagged sediment input became significant variables. Thus, shoreline position patterns in the study area were shown to be related to multiple factors, and the importance of these factors changed with distance along the beach.

## Discussion

We have shown that a burned California watershed generated elevated fluvial sediment discharge to the coast after post-fire storms that, in turn, expanded beach widths and resupplied the littoral cell with sand. However, it is also clear that not all wildfires exhibited similar responses. For example, consider the differences between the 2008 Basin Complex fire and the 2016 Soberanes fire (Fig. 2). Although the 2008 wildfire burned a larger portion of the Big Sur River watershed, it was followed by moderate rainfall conditions and only ~0.18 Mt of suspended-sediment discharge over three years (tenfold less than the event studied here), and the shoreline response was considerably muted compared to 2016–2020 (Fig. 2). Thus, similar wildfires can result in markedly different sediment yield and coastal responses.

The added effects of wildfire on watershed sediment yield during 2016–2017 were estimated to be a factor of 30-fold over what was expected given the year’s hydrologic conditions (Fig. 2). Without these wildfire effects, annual suspended-sediment discharge would have been only ~ 0.07 Mt, which is approximately the mean annual sediment discharge of the 50-year record. Over the actual 50-year record, however, the two years with the greatest wildfires effects combined to contribute 70% of the total river sediment discharge (Fig. 2). Thus, wildfire has the potential to be a dominating factor for sediment budgets for coastal watersheds like the Big Sur River, but only if the post-wildfire hydrologic conditions are adequately wet to induce enhanced erosion processes throughout the landscape^1,4,14,49^.

In addition to the implications of new sediment discharged to the coast, it is also important to note that the lack of sediment discharge during drought conditions, such as during 2012–2015, corresponded with some of the narrowest beach conditions on record (Fig. 2). As such, it appears that the lack of sediment during dry intervals can be similarly important to beach widths as the abundance of sediment after massive inputs. We hypothesize that these sediment-supply-dictated responses of the beach occur because sediment is transferred downcoast through the littoral system by northwesterly waves over time scales of one to several years (Fig. 4 and Fig. 6f–i).

**Figure 7:**
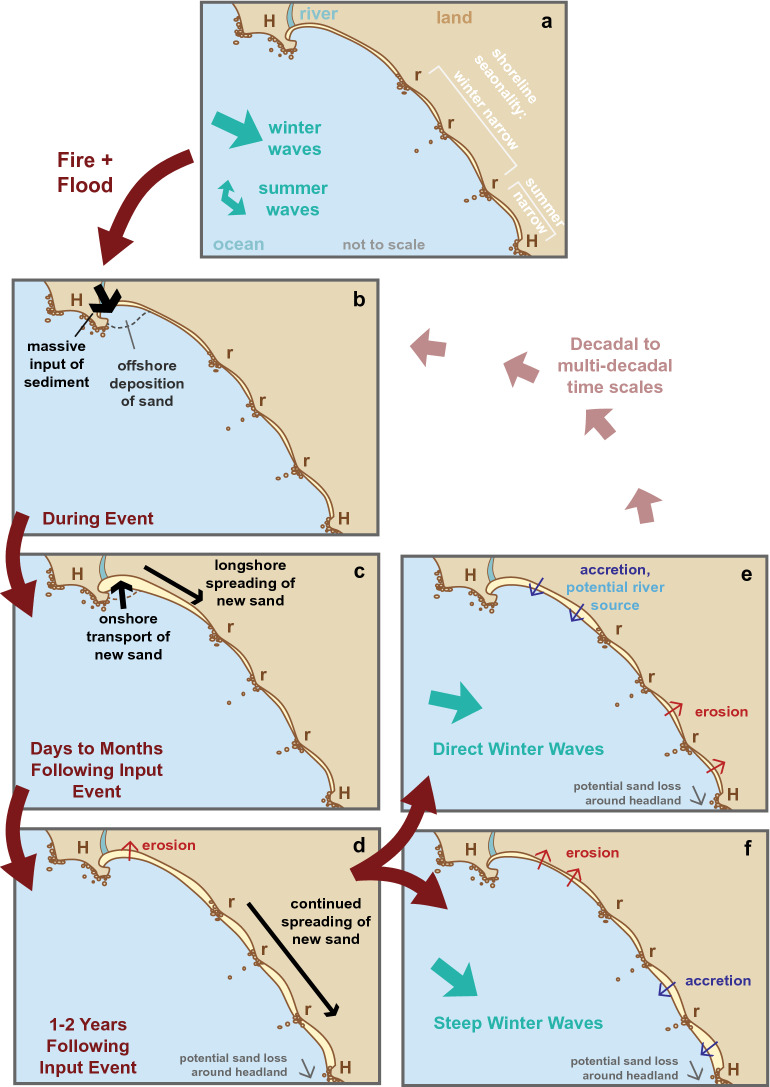
Conceptual model of the Big Sur River mouth littoral cell response to sediment input and wave conditions. Shoreline morphodynamics highlight changes to sandy beach widths (yellow areas) along a coast that includes two headlands (H) and several rocky areas (r).

A simple event-response conceptual model for the Big Sur River study site was constructed to summarize these findings (Fig. 7). For the Big Sur River setting, the seasonality in waves—larger, northwesterly waves in the winter and smaller waves with a southern component in the summer—provides fundamental forcing on this coast (Fig. 7a). This generally transports beach sand toward the south, where multiple rocky areas and a small headland interrupt sediment transport and form several ~ 400 m shoreline cuspate features. Additionally, seasonality in wave conditions causes seasonality in shoreline positions, especially in the central and southernmost section of the beach (Fig. 7a).

During fire-flood event sequences, the majority of newly introduced fluvial sediment will be deposited immediately offshore of the river mouth^47^, and much of it will move back inshore toward the beach and spread downcoast over weeks to months (Fig. 7b,c). Although the longshore spreading of this new sediment is initially slowed by the first rocky coastal section, it passes into the ~ 2-km littoral cell over the coming years as sediment is transported in the downcoast direction (Fig. 7d). This downcoast spreading of sediment occurs coincidentally with erosion of the broad beach at the river mouth, a geomorphic pattern that is conceptually similar to patterns found at many deltas worldwide that redistribute sediment from the river mouth toward distal regions of the delta^50^. It is likely that sediment is transported around the southern headland, and presumably lost to downcoast littoral systems, although we did not evaluate this hypothesis with our data (Fig. 7d).

Over the years following a fire–flood event, two endmember shoreline conditions are observed (Fig. 7e,f). During winters with more direct (westerly) wave approach, the beach is generally wider in the north and narrower in the south (Fig. 7e). The widening may be a function of differences in littoral sediment exchanges^51^ or from additional river inputs of sediment. Precipitation is notably increased during storms from more westerly directions^52,53^, suggesting that increased fluvial sediment discharge likely coincides with winters with more direct waves (Fig. 7e). Other winters are dominated by more northerly, or “steep” winter waves, which will cause the beach to be narrower in the north and wider in the south (Fig. 7f). Both of these post-event scenarios are hypothesized to transport littoral sediment around the southern headland, resulting in a gradual loss of sediment from the littoral system—and an overall narrowing of beach—over time (Fig. 7e,f).

Combined, these observations and models can be used to hypothesize how similar coastal systems will respond to climate change. For example, climate change will alter the distributions of many of the forcing factors found to be important to the system studied here, including the size, intensity, and frequency of wildfire, precipitation intensity during wet conditions, and the prolongation and intensification of drought between wet years^2,11,12,54^. In general, these changes are expected to increase sediment production to fluvial systems in the U.S. West^11^, which may result in more river sediment discharge events such as those detailed here and by others^42,47^. However, these fire and flood events will likely be separated by more intense drought conditions with negligible river sediment discharge^9,54^. Combined, this would lead to increased variability in fluvial sediment supply, and our observations suggest that this may result in increases in shoreline variability, such as during 2010–2020 at the study site (Fig. 2).

Because beaches provide important habitats, recreational opportunities, and storm hazard mitigation, it is crucial to better understand changes to these systems^23,26,55^. To encourage these studies, we note that publicly available satellite imagery collected during the past four decades was exceptionally useful for characterizing changes in this small littoral system with narrow beaches. This finding suggests that these tools can be applied broadly to develop a greater understanding of coastal morphodynamics and the physical factors that control margin-scale coastlines. Furthermore, broader application of satellite-based observations will allow for more examples of sediment supply events to coastal systems—from both fire–flood events and other events such as floods and landslides^42^—to expand the understanding of how landscape disturbances influence the world’s shorelines.

## Methods

### Satellite-derived shorelines

Shorelines were generated using the CoastSat open-source Python toolkit^36^, which utilized 1984–2020 Landsat and 2015–2020 Sentinel-2 imagery. The CoastSat algorithm combines a supervised classification and sub-pixel resolution border segmentation to map the synoptic boundary between sand and water in each cloud masked and pan-sharpened image. Shorelines were transferred to cross-shore positions along transects spaced at 100 m intervals and corrected for the effects of the local tide from a global tide level model and a computed beach slope for each transect^56^. The uncertainty in the shoreline positions resulting from these techniques is approximately 10 m^18,36,56^. Data used here are available from the CoastSat portal (http://coastsat.wrl.unsw.edu.au/) as Sites 163 and 162 for California. Sites 165 and 164 were added for the regional comparison shown in the Supplemental Materials.

Shoreline metrics were computed for each CoastSat transect. Annual values (median, quartile, minimum, and maximum) were computed over ‘water years’ (Oct. 1 to Sept. 30, and named for the calendar year in which it ends). The standard errors of annual median values were calculated by the ratio of 1.253 times the standard deviation of the annual values and the square root of the number of observations; and these were generally ~ 3.0 m with a standard deviation of 1.1 m, suggesting that differences in excess of 4–8 m were greater than the sampling uncertainty.

To examine the shoreline response to the 2016–2017 fire–flood event, the 2014–2020 data were divided into ‘before’ and ‘after’ intervals by a separation date of Mar. 1, 2017 and renormalized by subtracting the mean position of the ‘before’ interval. Mean and standard deviation positions for the intervals were compared with Welch’s t-tests, all of which indicated that the beach was significantly wider after the fire–flood event (all *p* < 0.0001).

To examine the initial timing of accretion, the 2014–2020 data were fit with locally estimated scatterplot smoothing (LOESS)^57^ functions using a fitting value (*f*) of 0.1. The maximum difference in the LOESS values was found for each transect, and the initiation of accretion was defined as the first date that a specific percentage of this difference occurred. Values from 33 to 90% showed similar patterns, so for simplicity the timing of the 50% values, which are referred to as the midpoints, are shown in the paper.

### Hydrologic data

Precipitation data were obtained from the monthly National Weather Service rainfall gauge at Big Sur (station BGS; Fig. 1), which are available at the California Data Exchange Center (CDEC; http://cdec.water.ca.gov/). Monthly data from the BGS station were summarized into water year increments. River discharge data were obtained from the15-min observations from USGS streamgage 11143000 (Fig. 1) available from the USGS NWIS system^58^. Additionally, the annual peak flow vales from 1950 to 2020 were used to compute annual exceedance probabilities of 0.5 and 0.1 (2- and 10-year recurrence values).

Fluvial sediment discharge estimates were derived by extrapolating from an adjacent watershed, the Arroyo Seco, because the Big Sur River does not have a suspended-sediment sampling program. The Arroyo Seco drains a similarly steep and forested landscape and shares over 16 km of ridgeline with the Big Sur River. River suspended-sediment monitoring on the Arroyo Seco includes years without wildfire impacts and two watershed-scale wildfires (1977 and 2008), which burned 100 and 93% of the watershed drainage area, respectively. From these data, Warrick et al. (2013) developed an annual sediment yield model based on precipitation, wildfire extent and time since wildfire^4^:

1 $${Q}_{ss}=m{P}^{n}{F}_{f}\left(t\right),$$

where *Q*_*ss*_ is the annual suspended-sediment discharge (Mt/year), *P* is the annual precipitation at station BGS (cm/year), *m* and *n* are coefficients derived from least-squares regression for years without wildfires effects (*m* = 7.52 × 10^−13^; *n* = 4.96, r^2^ = 0.80), *F*_*f*_ is termed the ‘*Fire Factor’* and represents the increase in suspended-sediment discharge that occurs from the cumulative wildfire effects in the watershed during year, *t*. The *F*_*f*_ is computed annually by summing the effects of previous wildfires:

2 $$F\left(t\right)=\sum_{all \; i}\frac{{A}_{i}}{{A}_{ws}}C{e}^{-k(t-{t}_{i})}$$

where, *A*_*i*_ is the area burned for fire *i*, *A*_*ws*_ is the total watershed area, *C* is a non-dimensional sediment yield increase factor that includes precipitation enhancement based on regression analyses^4^:

3 $$C=46.5\left(0.0101P-0.4957\right),$$

and the exponential function in Eq. (2) represents the decay in the wildfire effects with time from the year of the wildfire (*t*_*i*_). The decay coefficient (*k*) was found to be equivalent to a 1.4-year half-life (*k* = 0.5062) using post-fire records^4^. After 8 years transpire after a wildfire, it is excluded from Eq. (2), and *F*_*f*_ are not allowed to drop below values of unity, even during the driest conditions. The standard deviation of the lognormal output of this model is a factor of 2; that is, 68% of the estimates fall within approximately one half to two times the measured values.

For the Big Sur River watershed, values for *F*_*f*_(*t*) were derived from the wildfire perimeter maps provided by the California Department of Forestry and Fire Protection’s Fire and Resource Assessment Program (FRAP; https://frap.fire.ca.gov/mapping/), which provides data for all fires greater than at least 0.4 km^2^. The computed annual sediment discharge estimates from Eq. (1) were scaled from the Arroyo Seco (293 km^2^) to the Big Sur River watershed (151 km^2^) using the watershed area ratio (0.515).

### Cliff erosion

Cliff erosion is another primary sediment source to California beaches, including the study area (Fig. 1e). To estimate the volume of sediment contributed by cliff erosion, we utilized cliff erosion measurements for the study area from the USGS National Assessment Project, which suggest that approximately 0.3 m/year of cliff erosion occurred between 1933 and 1998^43^. For the study area, approximately 1700 m of the shoreline is backed by cliffs, and the cliff ranges from 20 m high in the north to 30 m high in the south. Using an average cliff height of 25 m, the average volumetric erosion can be estimated by the product of the three terms (height, length and erosion rate) to be approximately 12,500 m^3^/year. The lower half of the cliff material is weak metasedimentary rock from the Franciscan Formation, and the upper half is unconsolidated Quaternary materials, so an average bulk density of 2000 kg/m^3^ is assumed. Combined, this calculation suggests that about 25,000 t/year of rock and sediment are eroded from the cliff to the beach. We can compare the cliff sediment flux with the average river input (4.8 Mt over 50 years, or 96,000 t/year). Thus, using average rates, the Big Sur River provides ~ 80% of the sediment input to the study area, whereas cliff erosion provides ~ 20%.

### Oceanographic data

Wave observations were obtained from the National Data Buoy Center’s (NDBC) Cape San Martin buoy (Station 46,028; 35.770° N 121.903° W) using the NDBC data portal (https://www.ndbc.noaa.gov/). Monthly frequency and seasonal histogram plots (Fig. 1) were derived from hourly directional wave measurements during 1983–2020 of significant wave height (*H*_*s*_) and direction from which the dominant period was derived (*Dir*). Unfortunately, wave measurements were not continuous for the 1984–2020 satellite records. Thus, we utilized hindcast data from the ERA5 of the Copernicus Climate Change Service (C3S) to provide a continuous wave record to compare with the satellite-derived shorelines. Wave data were interpreted from the ERA5 records for the location of NDBC 46028 for all annual indices generated as noted below.

### Regression analyses

Annual median shoreline positions were used in single and stepwise multiple regressions to evaluate whether environmental factors may have influenced shoreline positions. Several independent variables were evaluated. For river sediment discharge, we utilized the annual data in two manners: (1) sediment discharge during the same year as the shoreline measurement, and (2) sediment discharge during the previous year. Other time lags were used (2–5 years) to see if the dominant shoreline response was longer than 1 year, but all resulted in lower correlation coefficients. Regressions were conducted with raw and log-transformed sediment discharge data, and both produced similar correlation coefficients. We present the log-transformed data owing to the likelihood of a non-linear response and to the strong skew in the sediment discharge data.

For waves, we used a similar approach as Barnard et al.^59^, who found that shoreline change in the broader region was related to winter storm wave energy flux and direction. Thus, we calculated the wave energy flux (*F*_*wave*_) from each hourly ERA5 hindcast value:

4 $${F}_{wave}= \frac{\rho {g}^{2}{{H}_{s}}^{2}{T}_{av}}{64\pi }$$

where ρ is the density of seawater (1025 kg m^−3^) and *g* is the acceleration of gravity. The fall-to-winter *F*_*wave*_ was calculated by summing over Oct. 1 to Mar. 30^59^, which was normalized by dividing by the average of all annual values. Because the direction of the most powerful waves also plays an important role in shoreline position^60,61^, we computed an average storm-season *Dir* by considering hourly records within the top 5% of each annual *F*_*wave*_ value. A compilation of these wave variables are presented in the Supplemental Materials.

Variables were tested independently in single variable linear regressions with the annual shoreline positions for each transect. The stepwise multiple regression was run with an additional independent variable, the median position of the shoreline during the previous year, to incorporate the potential for year-to-year ‘memory’ in shoreline position. Stepwise analyses were conducted in a forward manner for which the most significant variable was added by selecting the lowest *p* value at each step and limiting variables to *p* < 0.05.

## Supplementary Information

Supplementary Information 1.

## Data Availability

All data are publicly and freely available as noted in the “Methods” section and the references cited therein. The satellite-derived shorelines data for the Big Sur region can be visualized at http://coastsat.wrl.unsw.edu.au/ (sites usa_CA_0163 and usa_CA_0162).

## Code availability

The source code to map satellite-derived shorelines from Landsat and Sentinel-2 imagery (CoastSat) is available at https://github.com/kvos/CoastSat (https://doi.org/10.5281/zenodo.2779293). The source code to estimate beach slopes from satellite-derived shorelines and modelled tides (CoastSat.slope) to apply the tidal correction is available at https://github.com/kvos/CoastSat.slope (https://doi.org/10.5281/zenodo.3872442).
