# Supplementary material for: Fire (plus) flood (equals) beach: coastal response to an exceptional river sediment discharge event
Source: Sci Rep. 2022 Mar 9;12:3848. doi: 10.1038/s41598-022-07209-0 (PMC8907308; doi:10.1038/s41598-022-07209-0)
Supplement: Supplementary file 1 — Supplementary Information 1. [file 41598_2022_7209_MOESM1_ESM.pdf]

Supplemental Materials

Fire (plus) flood (equals) Beach: Shoreline response to an exceptional river sediment discharge event

Jonathan A. Warrick, Killian Vos, Amy E. East, Sean Vitousek

Scientific Reports

25th January 2022

INTRODUCTION

This Supplemental Materials section provides additional information about the shoreline change patterns in the broader region of the study area and provides more background information such as figures with the complete set of transects and the wave parameters used in the regression models.

REGIONAL SHORELINE CHANGE PATTERNS

To evaluate whether the shoreline change patterns observed near the Big Sur River mouth were unique for the broader region, we compared these data with other CoastSat data in the immediate region. Our approach was to compare broad patterns and trends across these sites, so we developed and compared site-averaged records for four locations near the study site. Two sites occurred north of the Big Sur River mouth, the beach north of Point Sur and a pocket beach between Point Sur and the river mouth that we term Swiss Canyon (***Supp. Fig. 1a***). These beaches are the first two sandy beaches north of the Big Sur River that are included in the

CoastSat database and are defined as Sites 165 and 164, respectively. The other two sites represent the broader Big Sur River mouth beach that was used in the paper (i.e., transects 100-1800 m). These sites are defined in CoastSat as Sites 163 and 162, which we term Big Sur River mouth and Franciscan Rocks, respectively, and represent transects 100-600 m and 700-1800 m. The separation point between these CoastSat sites is the first rocky region downcoast of the river mouth, which forms a natural boundary between river-dominated section to the north and the mixed influence region to the south (*e.g., see Discussion and Fig. 5 in paper*).

These CoastSat sites represent three beaches with little direct fluvial sediment contributions (Sites 162, 164, and 165) and one at the mouth of one of the region's largest watersheds (Site 163). Additionally, the sites differ markedly in their orientation and morphology. The Point Sur beach is bound by a southern headland, oriented toward the west-northwest, and backed by a vegetated dune field that extends southwest toward the downcoast shoreline. In contrast, Swiss Canyon (Site 164) is a pocket beach with rocky sections, oriented toward the west-southwest, and backed by an eroding bluff. These conditions at Swiss Canyon are most similar with the setting of Franciscan Rocks (Site 162). Lastly, the Big Sur River mouth (Site 163) is defined by the northern headland, Molera Point, is oriented toward the south to southwest, and receives fluvial inputs on its northern end.

For each of these four sites, raw CoastSat shoreline positions from multiple transects were combined into a single shoreline record. The total number of transects per site were 12, 6, 8 and 15 for Sites 162, 163, 164 and 165, respectively. To generate these shorelines, the raw CoastSat data were first normalized by subtracting the mean measured shoreline position at each transect. This normalization allowed for direct comparisons between transects, because the units

were converted from meters from an arbitrary landward point to meters from the mean shoreline position.

To combine the multiple transects into a single record for each site, the incompleteness of records was assessed. All transects had some records without data, and these data gaps could be attributed to conditions such as patchy cloud cover or haze in the imagery or incomplete satellite data coverage. For example, for the 1326 satellite images that captured some shoreline data for Site 162, only 920 (69.4%) returned shoreline data for every transect, whereas 1279 (96.5%) returned shoreline data for at least half of the transects. This situation was worst for Site 163, where patchy fog conditions were regular. For this site, a total of 1137 satellite images captured some data, but only 223 (19.6%) of these returned data for every transect, whereas 1005 (88.4%) returned shoreline data for at least half of the transects. To reduce the detrimental effects of computing mean values from only one or a few transects, while attempting to ensure a thorough time series for each site, we chose to include any mean record that had measurements for 50% or more of the transects within the site. This approach resulted in a total of 1279, 1080, 1068 and 1005 mean records for Sites 162-165, respectively. Mean shoreline position values for each of these records are shown in ***Supp. Fig. 2***.

The mean shoreline records for each of the four sites were markedly different. For example, the Point Sur shorelines recorded strong (40-60 m) seasonal cycles, Swiss Canyon and Franciscan Rocks recorded more moderate (20 m) seasonal cycles, and the Big Sur River mouth had negligible seasonal cycles (***Supp. Fig. 2***). Over multi-year intervals of time, each site recorded times with wider beaches and narrower beaches, and these patterns were not always coherent across the sites. For example, the Point Sur beach was relatively wide during 2012-2016, which is the same time that the Big Sur River mouth beach was relatively narrow (***Supp.***

Fig. 2a,c). Additionally, only the Big Sur River mouth site showed the pulsed increase in beach width during early 2017 (***Supp. Fig. 2***), which resulted from fluvial input as noted in the body of the paper.

Linear regression between the coincidental shoreline measurements of the four sites provides additional information about these temporal patterns. The correlation coefficients between the three beaches with little direct fluvial influence were all positive (r = 0.40 to 0.57; yellow lines; ***Supp. Fig. 1b***). In contrast, the correlation coefficients between the river mouth beach and the upcoast beaches were much lower (r = -0.09 to 0.05; white lines; ***Supp. Fig. 1b***). There was stronger correlation between the river mouth beach and the downcoast Franciscan Rocks beach (r = 0.46; white line; ***Supp. Fig. 1b***), which is consistent with the sediment transport connections discussed in the body of the paper.

Multi-year trends can also be assessed with the comparisons of annual shoreline metrics. For this, we have used the water-year approach, which defines years as Oct. 1 to Sept. 30 and is consistent with work in the body of the paper. Annual median shoreline positions for the four sites show that these values varied by meters to 10s of meters per year (***Supp. Fig. 3***). Linear regressions through these data show that the three beach sites are all positively correlated, albeit with r values of 0.18 to 0.26 (***Supp. Fig. 1c***). The river mouth site, in contrast, is negatively correlated with the northern beach sites (r = -0.27 to -0.39) but positively correlated with the southern Franciscan Rocks beach (r = 0.22; ***Supp. Fig. 1c***).

Additionally, these annual median shoreline positions can be compared using regression analyses with estimates of river sediment discharge and the storm-season wave conditions. River sediment discharge was observed to negatively correlate with the three beach sites (r = -0.13 to -0.56), but positively correlate with the river mouth site (r = 0.70; ***Supp. Fig. 1d***). If a 1-yr lag is

included in the river sediment response, the northern beaches still had negative correlations (r = -0.29 to -0.47), while the river mouth and southernmost beach had positive correlations (r = 0.32 to 0.42; ***Supp. Fig. 1d***). This correlation suggests that fluvial inputs of sediment were likely of little importance to the northern beaches, whereas the river mouth and southern beaches were wider following river sediment inputs as noted in the body of the paper. In fact, the negative correlation between river sediment discharge and the northern beach positions may be coincidental, owing to the co-occurrence of larger and more westly waves during wet years with higher discharge, as noted below.

Storm-season wave parameters, which included normalized fall-winter wave energy flux and the mean direction of the most powerful wave conditions (see Methods and additional information below), were compared to annual shoreline positions using linear regression. These analyses revealed that the three beach sites all were negatively correlated with wave power (r = -0.31 to -0.61), whereas the river mouth was positively correlated with wave power (r = 0.21; ***Supp. Fig. 1e***). This correlation suggests that the beaches were generally narrower following winters with large wave power, whereas the river mouth was somewhat wider. Correlations followed the opposite pattern with respect to the mean direction of the largest waves, which were positive for the beaches (r = 0.27 to 0.47) and negative for the river mouth (r = -0.11; ***Supp. Fig. 1e***).

Overall, these comparisons between the four sites suggest that the Big Sur River mouth site functioned in different ways than the other beach sites. Whereas the shoreline positions of the three beach sites were all positively correlated with each other, the shoreline positions of the river mouth exhibited different patterns (***Supp. Fig. 1***). Furthermore, the river mouth beach generally exhibited correlations that were in the opposite direction with respect to river sediment

discharge and wave conditions than the other beaches. That is, the comparisons and analyses here provide evidence that the beaches near the Big Sur River mouth were fundamentally different than other beaches in the area, and that river sediment inputs could explain some of these differences.

ADDITIONAL DATA PLOTS AND INFORMATION

Here we provide additional background data to those presented in the body of the paper. Two figures in the body of the paper were limited to only a few of the 18 transects of the study area. These figures included the annual shoreline metrics (median, quartiles, minimum and maximum; **Fig. 2** of the paper) and the raw and filtered shoreline position data focused on the 2014-2020 interval to provide further details of the shoreline responses to the fire-flood event (**Fig. 3** of the paper). These two figures are reproduced here with the complete set of study area transects (100 m to 1800 m; **Supp. Fig. 4 and 5**).

Additionally, we provide the time series of the annual wave parameters derived from the ERA5 hindcast of global ocean conditions. As noted in the Methods section of the body of the paper, these parameters included the normalized wave energy flux during the fall-to-winter seasons (Oct. to March) and the average direction of the most powerful waves, as defined by the upper 5% of the wave energy flux conditions. Annual time series of these parameters are shown in **Suppl. Fig. 6**. Additionally, these annual values are compared in a scatter plot to show the pattern between them, in which larger wave energy fluxes are generally related to more normal wave incidence angles (**Suppl. Fig. 6**).

Lastly, scatter plots between shoreline positions at two transects (100 and 1800 m) and the sediment discharge and wave parameter variables are included to provide additional

information about the single regression analysis results found in the body of the paper (**Supp. Fig. 7 and 8**). For example, these plots show the positive correlation ($r = 0.74$) between annual median shoreline positions at transect 100 m and the annual sediment discharge from the river and the positive correlation ($r = 0.41$) between the shoreline positions at 1800 m and the 1-yr lagged sediment discharge (**Supp. Fig. 7**). Additionally, these figures show how the correlations with wave parameters at transect 100 m are in the opposite direction to those at transect 1800 m (**Supp. Fig. 8**).

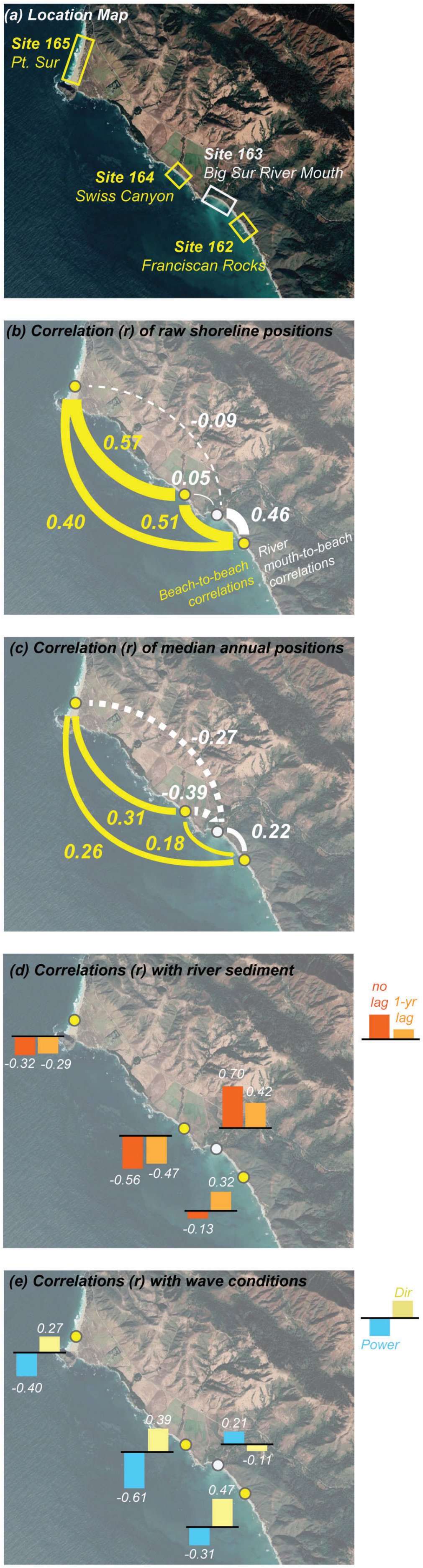

Supplemental Figure 1. Comparisons of CoastSat shoreline data for the broader region near the Big Sur River mouth. (a) Location map of the four CoastSat sites compared in this analysis. (b-c) Correlation coefficients (r) for linear regressions between the (b) raw and (c) annual median CoastSat data from the four sites. Comparisons with the Big Sur River mouth are shown in white, comparisons between beaches are shown in yellow. The thickness of the lines are proportional to the *r*-values. Solid lines represent positive correlations, and dashed lines are negative correlations. (d-e) Correlation cofficients (*r*) for linear regressions between the annual median shoreline positions and (d) estimated annual sediment discharge from the Big Sur River, both without a lag (dark orange) and with a 1-year lag (light orange), and (e) storm-season wave parameters including the annual fall-winter wave energy flux (blue) and the mean direction of the largest waves (yellow). See Methods for descriptions.

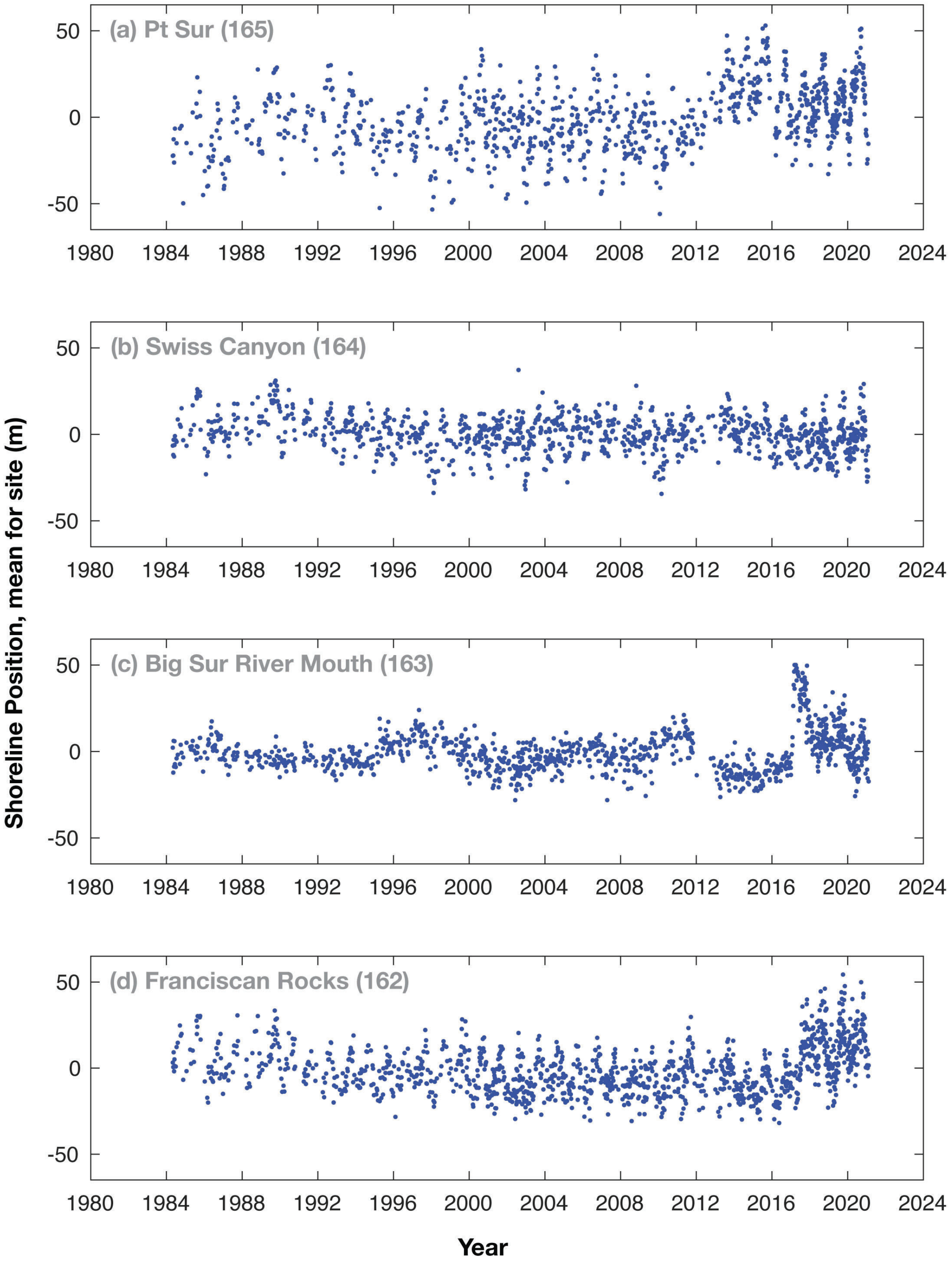

Supplemental Figure 2. Mean shoreline position records for each of the four primary CoastSat areas near the Big Sur River mouth. See text for averaging methods.

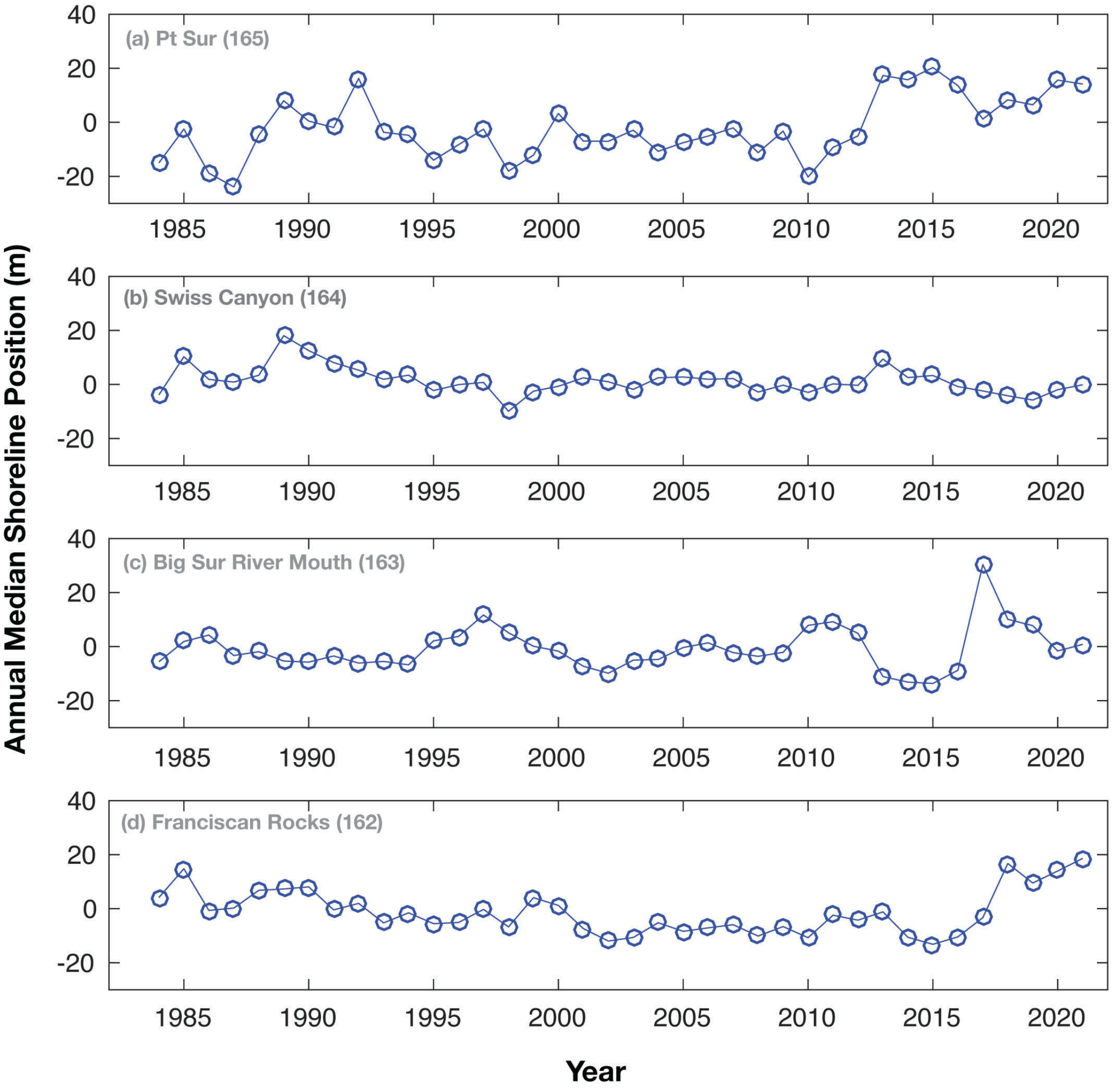

Supplemental Figure 3. Comparison of the annual median shoreline positions for each of the four CoastSat areas near the Big Sur River mouth. See text for computational methods.

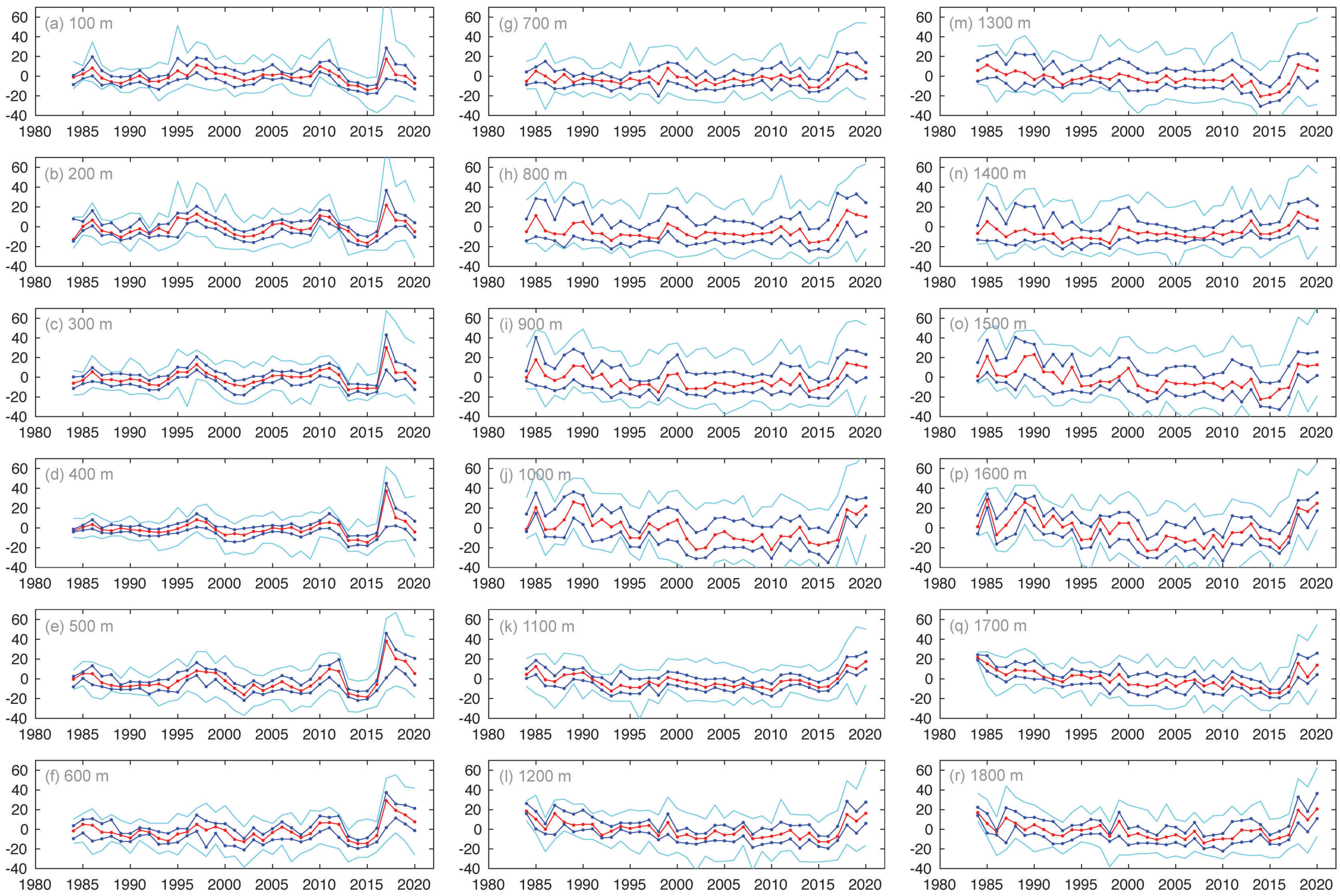

Supplemental Figure 4. Annual water year (Oct .1 to Sept. 30) shoreline positions from CoastSat for each of the Big Sur River transects. Annual data have been summarized into median (red symbols and lines), upper and lower quartiles (blue symbols and lines), and minimum and maximum (cyan lines).

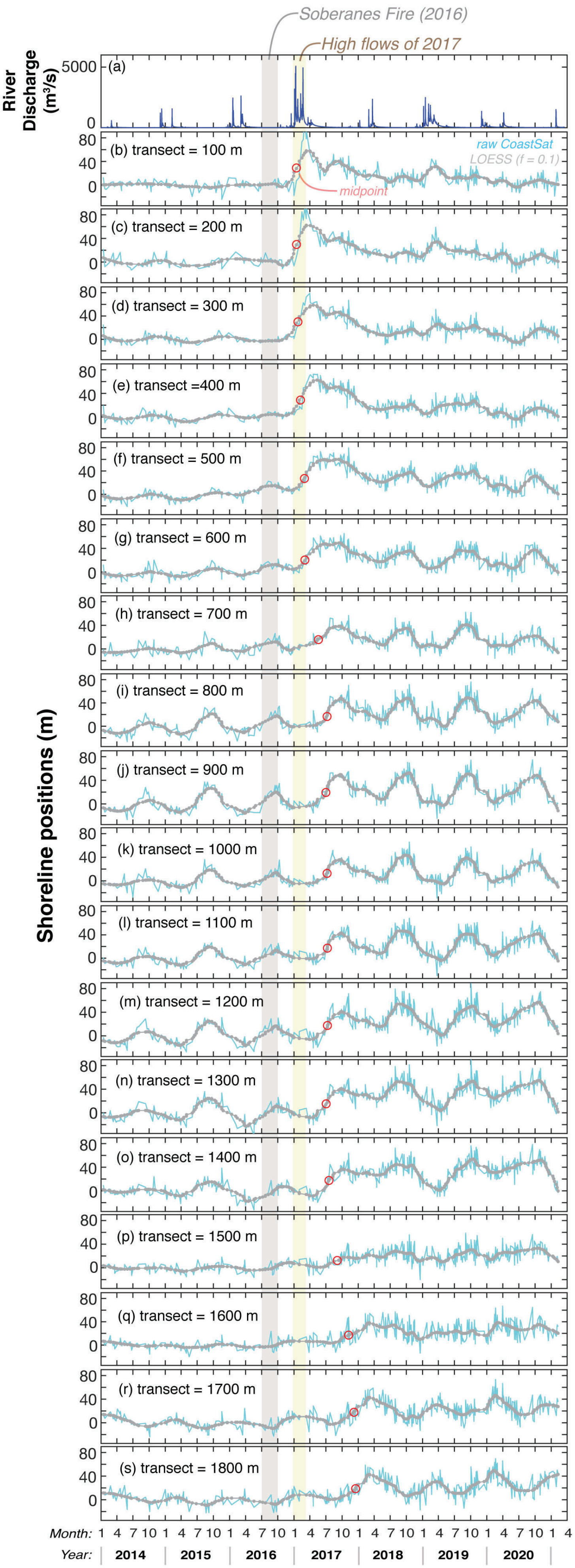

Supplemental Figure 5. The complete set of CoastSat transects for the Big Sur River mouth study area during 2014-2020. Data include raw shoreline position (light blue) and LOESS fit (grey) through the raw data. The mid-point of the initial accretion signal is highlighted (red ciricles).

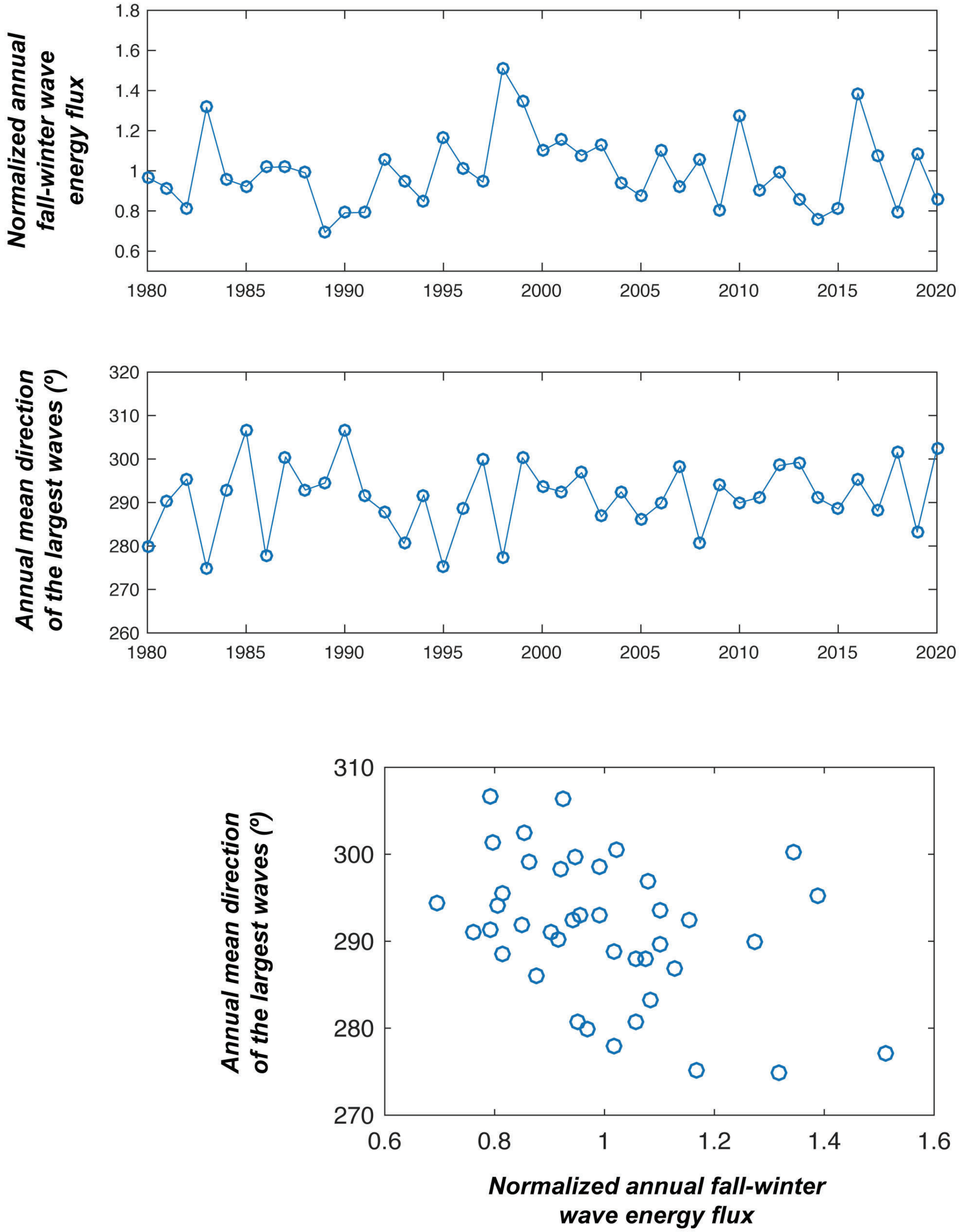

Supplemental Figure 6. Annual wave parameters calculated from the ERA5 hindcast sampled for the location of the NDBC San Martin Buoy (Station 46028). These data were used in the regression analyses. See Methods for descriptions of data.

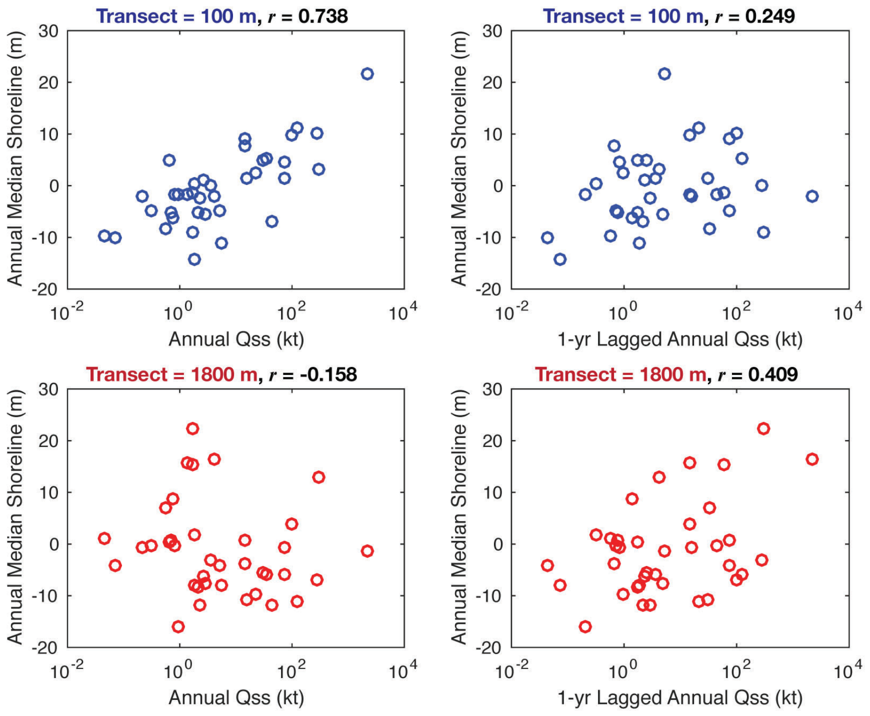

Supplemental Figure 7. Comparison of annual median shoreline positions for CoastSat transects at 100 m and 1800 m and estimated annual river suspended-sediment discharge (Qss) for the same year and the previous year (i.e., no lag and 1-yr lag).

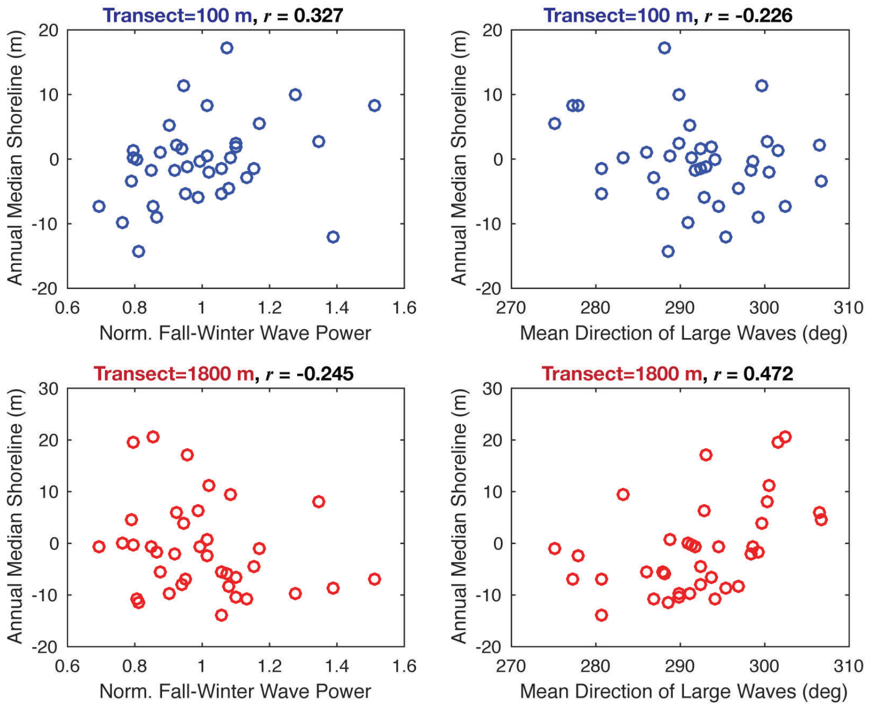

Supplemental Figure 8. Comparison of annual median shoreline positions for CoastSat transects at 100 m and 1800 m and two annual wave parameters: normalized fall-winter wave power and mean direction of the largest waves. See text for descriptions of wave parameters.
